# A Low‐Resource Intervention to Improve Emergency Medicine NBME Scores: Risk Notification and Study Plan Submission

**DOI:** 10.1002/aet2.70205

**Published:** 2026-06-01

**Authors:** Rachel L. Day, Nicholas Harrison, Malia J. Moore, Audrey Herbert, Anna Bona, Katie Pettit

**Affiliations:** ^1^ Indiana University School of Medicine Indianapolis Indiana USA

## Abstract

**Background:**

It is unclear what strategies are helpful to support medical students who are at risk of failing required clerkship final examinations, such as the commonly used National Board of Medical Examiners shelf exam (NBME). This study examined whether asking students who performed poorly on their practice Emergency Medicine NBME to create their own study plan, regardless of their chosen study method or resource, would result in better performance on the end of clerkship NBME.

**Methods:**

700 students enrolled in a required Emergency Medicine (EM) clerkship were evaluated during the Academic Year (AY) before and after implementation of the study plan requirement (AY‐2023 vs. AY‐2024). In both AYs, a practice NBME examination was required in addition to the mandatory end‐of‐clerkship exam, but in AY‐2024 students scoring 65 or below on the practice test were required to create a study plan without any specific resource or time‐commitment requirements. We compared the relationship between practice and final NBME scores pre/post intervention, using mixed modeling regression for multivariable adjustment of confounders.

**Results:**

After implementation of the study plan requirement for students failing the practice test in AY‐2024, both the overall NBME shelf exam scores and the rates of High Pass or Honors level final NBME scores rose. The difference in final exam scores between those failing the practice vs. passing it was dramatically smaller and no longer statistically significant between AY‐2023 and AY‐2024.

**Conclusion:**

For medical students that failed the Emergency Medicine NBME practice exam, a simple intervention of notification of potential failure and instruction to develop a study plan was associated with improved outcomes on the final examination, even without any requirement for the format or intensity of the study plan.

## 1 Introduction

The National Board of Medical Examiners (NBME) subject shelf exams are an important tool for measuring student learning during medical school clerkships and are a predictor of student success for the United States Medical Licensing Examination (USMLE) [1]. Significant time and resources have been utilized to determine how best to help students prepare for the subject NBME exams with prior studies examining whether specific study materials, the number of study materials used, or time spent studying correlate with better scores [2, 3].

While it is important for educators to direct students to effective study resources, it is unclear whether access to these materials alone or specific requirements to utilize these resources yield better exam scores. One prior study by Kastenmeier, et al. showed improved exam scores after implementation of individual learning plans on learner selected topics. However, these plans required use of a specific resource and completion of structured assignments around students' chosen topics [4].

Students completing their Emergency Medicine clerkship at our institution have a wide variety of study materials available to them, including multiple question banks, a suggested reading list, asynchronous lectures, and a required practice NBME exam. We hypothesized that asking students who performed poorly on their practice NBME to create their own study plan, regardless of their chosen study method or resource, would result in better performance on the end of clerkship NBME.

## 2 Methods

### 2.1 Population and Timeline

This IRB‐approved study was conducted within a single statewide medical school encompassing 9 regional campuses. We examined NBME shelf exam scores, mid‐clerkship practice NBME scores, and written mid‐clerkship study plans from all students completing a required 4‐week Emergency Medicine (EM) clerkship. Student data was extracted for two consecutive 1 year periods: academic year (AY) 2023 and AY‐2024. AY‐2023 included 338 4th year students and AY‐2024 included 362, for a total of 700 students analyzed.

### 2.2 Intervention

AY‐2023 and AY‐2024 were chosen for pre−/post‐ comparison to evaluate a curricular change that began in AY‐2024. In both years, students were required to take a practice NBME exam in the second week of their clerkship. However, starting in AY‐2024, students scoring 65 or below on this mid‐clerkship practice exam were required to write and report a self‐designed study plan to the clerkship leadership team. They were given no additional requirements or details to guide their study plan. There were no additional milestones or checkpoints prior to the final exam. Once submitted, the study plans were de‐identified and placed in a secure folder for research purposes. Students scoring above the cutoff of 65 on their mid‐clerkship practice exam had no additional requirements and were not required to file a study plan. Examples of actual student study plans are shown in the Appendix A. Prior to AY‐2024 (i.e., the AY‐2023 comparator group), students were required to take the practice NBME during mid‐clerkship but had no requirement for follow‐up or evaluation of the practice test's results.

### 2.3 Outcomes

The primary outcome compared between the pre‐ and post‐intervention cohorts (i.e., AY‐2023 and AY‐2024, respectively) was the students' scores on the end‐of‐clerkship NBME shelf exam (0–100 scale). As secondary outcomes, we also evaluated the rate of students achieving the binary NBME shelf score cutoffs of ≥ 73 (“High‐Pass”, HP). We hypothesized that students would have greater NBME shelf exam scores in AY‐2024, after implementation of the required study plan for low scores on the practice NBME test. Furthermore, we hypothesized that the greatest gains before and after the intervention would be seen among low‐scoring students (i.e., those subjected to a study plan in AY‐2024).

### 2.4 Data Analysis and Controlling for Confounders

We hypothesized that the month in which a student completed their rotation, the regional campus at which they completed their rotation, and the general relationship between practice exam and final exam score could be significant confounders. For timing, we speculated that NBME scores would vary based on seasonality. For instance, high rates of students interested in EM residency rotate during the summer months and could be expected to do better on the NBME shelf. Conversely, it is plausible that students rotating at the end of the academic year and after the Residency Match (e.g., March–April) could perform worse, due to feeling comfortable in their post‐medical school appointments. With regards to locations, medical students complete their required EM clerkship at any one of the medical school's 9 regional campuses. One campus is a large urban area with clinical rotations hosted at three level‐one trauma centers and is home to a PGY 1–3 EM residency, producing 21 EM physician graduates each year. By contrast, no other regional campus had an EM residency program or level‐one trauma center. Additionally, any students identifying an interest in EM residency in their early 4th year are moved to the urban/residency‐hosting campus to complete their rotation (i.e., regardless of which regional campus their other core/required rotations occurred at). Finally, we suspected that there would be an overall positive relationship between practice and overall NBME scores, at least in part due to factors beyond our intervention of interest. For instance, students who were better test takers or had more general medical knowledge would be expected to do better on both the practice and final NBME exams, regardless of the implementation of a study plan. Finally, we hypothesized that the effect of requiring a study plan for low‐scorers on the mid‐clerkship practice exam would only affect the scores of those low‐scorers, since those students above the “pass” threshold on the practice exam were treated no differently pre‐ vs. post‐ intervention.

To account for confounding on the above factors in our conceptual model, we employed mixed effects regression modeling. Two models were fit: a linear mixed model for the primary outcome (NBME numeric score), and a generalized linear mixed model with a binomial link for the secondary outcome (achieving/not‐achieving High‐Pass or Honors). In both models, random intercepts were fit nesting rotation month within site, and a random slope for the numeric practice exam score was included to account for correlation between practice exam and final exam scores unrelated to the intervention (as described above). Fixed effects in both models included the rotation year (AY‐2023 vs. AY‐2024) as a stand‐in for the intervention, along with whether or not the student “failed” the mid‐clerkship practice exam (i.e., score ≤ 65, triggering a self‐identified study plan in AY‐2024 but not AY‐2023). Since our primary hypothesis was that the intervention would have a moderating effect on the relationship between mid‐clerkship exam results and final NBME shelf exam score, an interaction term between year and mid‐clerkship exam pass vs. fail was included. Residuals were plotted, and model tests of fit were performed to evaluate model assumptions. All analyses were performed in R (version 4.3.0). An alpha of 0.05 was considered statistically significant.

Finally, we performed a qualitative review and analysis of AY‐2024 students' submitted study plans. This included a thematic analysis, with particular attention to whether the plans included additional study questions, readings, and/or additional practice tests. We also examined the specificity of the plan's timeline, e.g., whether a student detailed specific study activities by dates or rather simply listed the entirety of their plan to be completed before the final exam.

## 3 Results

### 3.1 Overall

Among 700 total students, the median (interquartile range, IQR) scores for the final NBME exam were 81 (76–85) and 80 (76–84) in AY‐2023 and AY‐2024, respectively. Mid‐clerkship practice NBME exam scores were 79 (73–86) and 81 (73–86) in the same years.

### 3.2 Qualitative Analysis

Of the 28 students in AY‐2024 that scored below the ≤ 65 threshold on the practice NBME, all were notified at the midpoint of their clerkship that they were at risk of failing the final NBME shelf and needed to submit a study plan. Of those 28, 19 of those students submitted a study plan to the clerkship leadership team, while 9 did not comply with the requirement. An overview of the thematic analysis is detailed in Table 1.

**TABLE 1:** Thematic analysis of submitted study plans (*n* = 19).

	YES (%)	NO (%)
Additional questions	18 (95)	1 (5)
Specific question topics identified	10 (53)	9 (47)
Readings	16 (84)	3 (16)
Additional practice test	11 (58)	8 (42)
Specific timeline identified	13 (68)	6 (32)

Study plan format varied between students. Some students submitted a schedule template while others submitted an outline or a paragraph description of the plan. Length of study plan in words ranged from 32 (one sentence) to 306. The most common item identified as part of a study plan was the intent to perform additional practice questions. 68% of the students submitted a plan with a specific timeline. Nine students did not submit the required study plan for unclear reasons. However, unlike in AY‐2023, these AY‐2024 students were still specifically notified by the clerkship leadership team of their low score and that they were at risk of failing the final NBME exam.

### 3.3 Primary and Secondary Analyses

**FIGURE 1:**
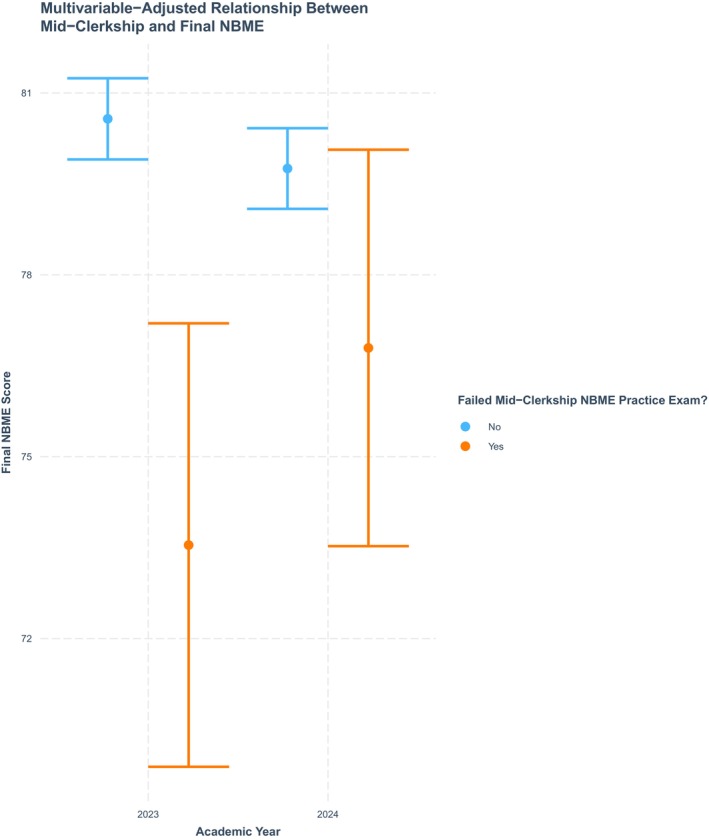
Multivariable‐adjusted relationship between mid‐clerkship NBME score and final NBME score.

The relationship between failing the mid‐clerkship practice NBME exam and final NBME exam scores, after multivariable adjustment for rotation month, site, and overall practice‐final exam score correlation, is presented in Figure 1. In AY‐2023, final NBME exam scores were significantly lower among those failing the mid‐clerkship practice NBME (73.5F, 95% Confidence Interval (CI) 69.7–77.4) than those exceeding the passing margin on the practice exam (80.6, 79.7–81.4). However, after implementation of the study plan requirement for students failing the practice test in AY‐2024, the difference in final exam scores between those failing the practice vs. passing it was dramatically smaller and no longer statistically significant (fail practice = 76.8 {95% CI: 73.4–80.2}; pass practice = 79.6 {78.9–80.6}).

**FIGURE 2:**
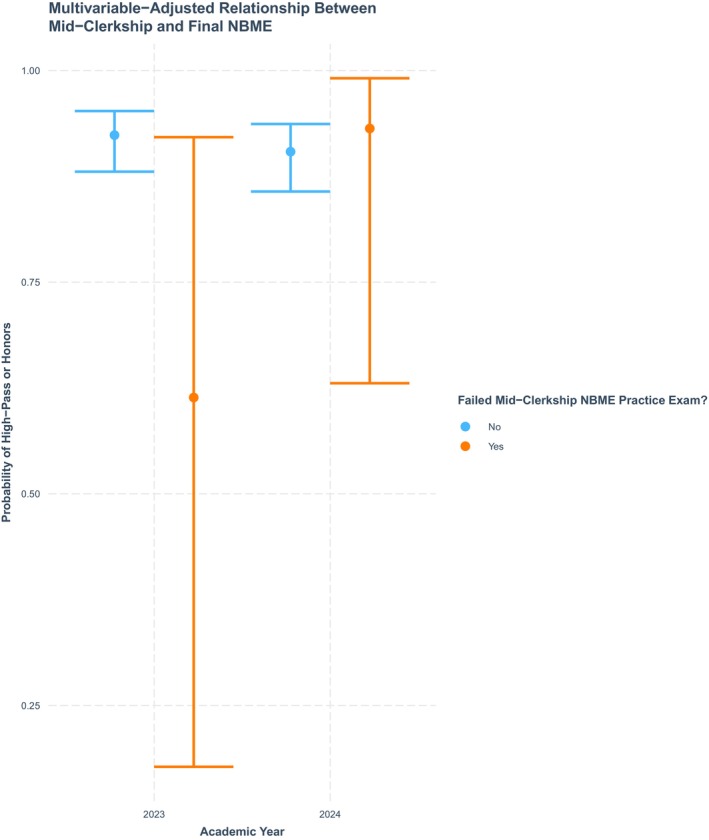
Multivariable‐adjusted relationship between mid‐clerkship NBME score and final NBME high/pass or honors.

The interaction between NBME practice exam pass/fail and pre/post‐ intervention (i.e., year) as it relates to the probability of achieving a “High Pass” (HP) or “Honors”‐level final NBME score is shown in Figure 2. In AY‐2023, the odds of achieving HP or Honors were significantly lower among students failing the mid‐clerkship practice exam (Odds ratio {OR} to achieve HP = 1.59; 95% CI: 0.22–11.7) as compared to students passing the practice exam (OR = 12.1; 7.4–19.9). Conversely, after study plan implementation in AY 2024, the odds of achieving HP were similar among those failing (OR = 13.6, 1.7–107.8) and passing (OR = 9.4, 6.0–14.9) the practice exam. When comparing just those students failing the practice exam, the probability of achieving HP on the final exam was also significantly higher overall in AY‐2024 compared to AY‐2023 (Figure 2).

## 4 Discussion

In this observational study of medical student NBME shelf exam scores, both overall NBME shelf exam scores and the rates of HP or Honors level NBME scores rose after a simple intervention instructing students who failed the NBME practice exam that they were at risk of failing and to develop a study plan.

Identifying at‐risk students early is important in medical education, and research has turned to machine learning to identify these students [5, 6]. This study suggests this can be done with one data point when targeting a specific outcome.

Undergraduate medical education uses remediation plans for many outcomes and phases of education with success [7]. To our knowledge, we have not seen published evidence that simply telling a student they will fail will improve their performance. Formal remediation plans are resource‐consuming for students as well as educators [8]. In fact, a recognized barrier to providing negative or failing feedback to learners in medical education is concerns about increasing the educator's own workload, and how to address that learner's specific deficits [9].

Interestingly, this effect was observed even without a specific requirement for type or format of the study plan, and without any faculty oversight of whether the student actually carried out their plan as proposed. These results could suggest that medical students, as adult learners, may benefit simply from the “wake‐up call” of being told by faculty when they are “behind” in their preparation for a written exam, even without close oversight of study and remediation. While certainly not all medical education outcomes can be treated as a single exam score, more research into how much motivation a simple notice of failure can provide could save time and energy.

In terms of the study plans themselves, most students turned to questions as the main resource to improve their knowledge and therefore exam scores. This is not surprising given previous research that practice questions are a high yield resource for medical students [10]. Given the significant increase we saw in their final exam scores, this would support inclusion of question‐based study materials for students at risk for failing exams.

Interestingly, 32% of the students that failed the practice test were notified of the failure but did not follow through with submitting a study plan. It is unclear whether these specific students scored lower than colleagues as the study plans were de‐identified. This further supports the possibility that the simple act of notifying a student that they are at risk to fail may be as important as the remediation plan itself.

## 5 Limitations

This study was performed at a single institution over a 1 year period. Additionally, this institution includes 9 regional campuses. This was performed only during the EM clerkship. Therefore, this finding may not be applicable or replicated in all specialties or across institutions. We purposely did not give any instruction on how to put together a study plan, what the study plan should include, or monitor progress. It is unclear whether that would have further improved exam scores.

## 6 Conclusion

For medical students that failed the Emergency Medicine NBME practice exam, a simple intervention of notification of potential failure and instruction to develop a study plan improved scores on the NBME shelf exam.

## Author Contributions

**Audrey Herbert:** conceptualization, writing – review and editing. **Katie Pettit:** conceptualization, writing – original draft, writing – review and editing, investigation, supervision. **Malia J. Moore:** conceptualization, writing – review and editing. **Nicholas Harrison:** conceptualization, writing – original draft, writing – review and editing, formal analysis, methodology, investigation. **Anna Bona:** conceptualization, writing – review and editing. **Rachel L. Day:** conceptualization, writing – original draft, writing – review and editing, investigation.

## Conflicts of Interest

N.H.‐ NH's institution has received grant funding from the National Institutes of Health, Doris Duke Foundation, and Indiana CTSI for investigator‐initiated research. NH's institution has received contract funding from Siemens, Beckman‐Coulter, Corteria Pharmeceuticals, and Abbott for industry‐initiated research. N.H. has received funding personally from Vave Health and EB Medicine for consulting and honoraria. The authors declare no conflicts of interest.

## Data Availability

The data that support the findings of this study are available from the corresponding author upon reasonable request.

## Appendix A

### Examples of Submitted Study Plans

*three examples given to reflect differing quality and detail of submission.

Example 1:“Appendix Example 1” table.

Example 2:

Thanks for reaching out and for your concern. I had not reviewed much of the subject material prior to the test as I wanted to see where I stood without it. I plan to do daily U‐world questions and to do the course materials on the Canvas page prior to the NBME. So I am confident I will be fine on the NBME.

Example 3:

Emergency Medicine Study Plan:

Tuesday, December 10th – Complete EM modules.

SAEM practice questions (40).

Wednesday, December 11th– SAEM practice questions (60‐80).

Review Neurology concepts.

Thursday, December 12th – Practice test.

Friday, December 13th – Review practice test. SAEM questions (60‐80).

Review cardiology concepts.

Review respiratory concepts.

Saturday, December 14th – SAEM practice questions (60‐80).

Review ID concepts.

Review psych concepts.

Sunday, December 15th – SAEM practice questions (60‐80).

Review OB concepts.

Review GI concepts.

Monday, December 16th – Practice Test.

Tuesday, December 17th – SAEM practice questions (60‐80).

Wednesday, December 18th – SAEM practice questions (60‐80).

#### Appendix Example 1

**Table:**

Date	UWorld	Canvas Module/Quiz	CDEM M4 curriculum	EM shelf review podcast
4/15	40 questions + review answers	Finish Cardiology, Respiratory, Healthcare/Billing		Listen to an episode while I drive, work out, cook, or clean
4/16	40 questions + review answers	Neuroloy, Sepsis + shock	Review cardiology cases	Listen to an episode while I drive, work out, cook, or clean
4/17	40 questions + review answers	Trauma, Endocrine	Review respiratory + neurology cases	Listen to an episode while I drive, work out, cook, or clean
4/18	40 questions + review answers	Psych, OB/GYN + GU	Review sepsis + trauma cases	Listen to an episode while I drive, work out, cook, or clean
4/19	40 questions + review answers	GI, Ultrasound	Review endocrine + psych cases	Listen to an episode while I drive, work out, cook, or clean
4/20	40 questions + review answers	Environmental/Toxicology, Ophthalmology	Review OB/GYN + GI cases	Listen to an episode while I drive, work out, cook, or clean
4/21	40 questions + review answers	RUSH	Review Environmental/Tox. + Ophtho cases	Listen to an episode while I drive, work out, cook, or clean
4/22	40 questions + review answers			Listen to an episode while I drive, work out, cook, or clean
4/23	40 questions + review answers			Listen to an episode while I drive, work out, cook, or clean
4/24	Exam day			
